# Lessons From the Polio Endgame: Overcoming the Failure to Vaccinate and the Role of Subpopulations in Maintaining Transmission

**DOI:** 10.1093/infdis/jix108

**Published:** 2017-07-01

**Authors:** Kimberly M. Thompson, Radboud J. Duintjer Tebbens

**Affiliations:** 1 Kid Risk and; 2 College of Medicine, University of Central Florida,Orlando

## Abstract

**Background.:**

Recent detections of circulating serotype 2 vaccine-derived poliovirus in northern Nigeria (Borno and Sokoto states) and Pakistan (Balochistan Province) and serotype 1 wild poliovirus in Pakistan, Afghanistan, and Nigeria (Borno) represent public health emergencies that require aggressive response.

**Methods.:**

We demonstrate the importance of undervaccinated subpopulations, using an existing dynamic poliovirus transmission and oral poliovirus vaccine evolution model. We review the lessons learned during the polio endgame about the role of subpopulations in sustaining transmission, and we explore the implications of subpopulations for other vaccine-preventable disease eradication efforts.

**Results.:**

Relatively isolated subpopulations benefit little from high surrounding population immunity to transmission and will sustain transmission as long as they do not attain high vaccination coverage. Failing to reach such subpopulations with high coverage represents the root cause of polio eradication delays. Achieving and maintaining eradication requires addressing the weakest links, which includes immunizing populations in insecure areas and/or with disrupted or poor-performing health systems and managing the risks of individuals with primary immunodeficiencies who can excrete vaccine-derived poliovirus long-term.

**Conclusions.:**

Eradication efforts for vaccine-preventable diseases need to create performance expectations for countries to immunize all people living within their borders and maintain high coverage with appropriate interventions.Keywords. Polio; eradication; transmission; heterogeneity.

Polio eradication continues to take longer and cost more than expected. The Global Polio Eradication Initiative (GPEI) currently hopes to interrupt wild poliovirus serotype 1 (WPV1) transmission by the end of 2017 or 2018 and stop all oral poliovirus vaccine (OPV) use in 2021 or 2022 [1]. Reaching the goal of polio eradication requires all countries to achieve and maintain high population immunity to transmission as long as polioviruses circulate anywhere [2]. The delays experienced by the GPEI provide a powerful reminder that eradication represents an unforgiving goal and that achieving eradication depends on addressing the weakest links [3].

As of the end of 2016, the GPEI continues to identify weak links after it is too late to prevent outbreaks. In March and August 2016, surveillance detected circulating serotype 2 vaccine-derived polioviruses (cVDPV2s) in Borno State (in northeast Nigeria) that genetically linked to a May 2014 cVDPV2 isolate from Borno that originally emerged in Chad [4]. These detections indicated that Nigeria and the GPEI failed to stop all persistent cVDPV2s prior to the globally coordinated cessation of the use of oral poliovirus vaccine (OPV) containing serotype 2 (OPV2) in late April and early May 2016, despite this representing a prerequisite to OPV2 cessation [5, 6]. The significant gap in access for immunization and surveillance activities in the area due to conflict occurred despite national efforts by Nigeria to perform trivalent OPV (tOPV) campaigns to boost population immunity to transmission prior to OPV2 cessation [7]. In late 2016, Nigeria also detected an unrelated cVDPV2 isolate in Sokoto (northwest Nigeria) [8], which indicated that its tOPV intensification efforts also failed to prevent the creation of new cVDPV2s. In early August 2016, the GPEI reported the detection of 2 polio cases caused by WPV1 in the same state of Borno [8]. This news disappointed hopes that the absence of reported WPV1 cases in Nigeria since July 2014 reflected the interruption of WPV1 transmission in Africa. The Borno WPV1 cases were most closely genetically related to isolates from Borno in 2011, indicating undetected circulation for 5 years. Considered together, these events strongly suggest that the lack of access to areas of Borno and other relatively inaccessible areas in neighboring states and countries in the Lake Chad Basin resulted in major gaps in both vaccination and surveillance. With the last reported polio case caused by WPV3 reported globally in November 2012 from an area near Borno (in Yobe) in Nigeria [9], poor-quality immunization and surveillance in this area raises concerns about the potential for undetected WPV3 circulation. In December 2016, the GPEI confirmed circulation of cVPDV2 in Balochistan Province, Pakistan [10], which confirms that Pakistan failed to perform sufficient tOPV campaigns prior to OPV2 cessation to prevent cVDPV2s [7]. With ongoing transmission of WPV1 in relatively inaccessible parts of Pakistan and Afghanistan [8], these areas continue to present significant challenges.

As we approach WPV eradication and manage OPV cessation, the vaccination of subpopulations emerges as critical for success. Undervaccinated subpopulations can sustain transmission and pose challenges because of a confluence of factors [11, 12] related to political circumstances (including poor program performance, low vaccination coverage, poor surveillance, and/or poor data quality), conditions that favor intense fecal-oral poliovirus transmission and correlate with low socioeconomic status (including poor sanitary and hygienic conditions, high birth rates and crowding, poor nutrition, poverty, and high exposure to pathogens that interfere with vaccine response), and/or limited access (including immigrants, displaced populations, and populations in violent, insecure, or remote areas) [13–21]. However, other subpopulations also emerge as important, including those in countries with sufficient access to vaccine who refuse immunization [22–26]. The lessons learned during the polio endgame about weak links and subpopulations should prove useful for future efforts to eradicate other vaccine-preventable diseases.

## METHODS

We reviewed the experience of the GPEI as of the end of 2016 to identify and model key subpopulations that delayed the achievement of polio eradication to date and that may threaten its long-term success. We performed a literature review of the Web of Science for articles published in English in peer-reviewed journals before 1 January 2017 with the key words “polio” AND “vaccine” AND “model” AND (“undervaccinated” OR “under-vaccinated” OR “subpopulation” OR “sub-population” OR “missed” OR “heterogeneity”). We also reviewed the references of these studies and considered the insights from modeling efforts that evaluated the polio endgame and long-term risks. Managing the risks of the polio endgame requires ending the use of OPV, to eliminate OPV-related risks (ie, vaccine-associated paralytic polio, which occurs in approximately 1 per million first OPV infections; cVDPVs, which can emerge in areas with low immunity and behave like WPVs; and cases involving rare individuals with primary immunodeficiencies who become infected with OPV and can excrete presumed fully transmissible and neurovirulent immunodeficiency-associated vaccine-derived poliovirus [iVDPV] for years) [27–29]. Risks after OPV cessation include the failure to use sufficient OPV before cessation or to synchronize OPV cessation globally, inadvertent use of OPV after OPV cessation, reintroduction of VDPVs (ie, cVDPVs and iVDPVs) created by exposures to OPV used prior to OPV cessation, and (un)intentional releases from vaccine manufacturing sites or laboratories [27–31]. This analysis complements work that focuses specifically on the lessons learned from globally coordinated OPV2 cessation [32].

We use an existing differential equation–based poliovirus transmission and OPV evolution model [33, 34] to illustrate the interaction between the size of an undervaccinated subpopulation, its degree of isolation from a well-vaccinated general population (ie, quantified by p_within_, which we define as the proportion of contacts of people inside the undervaccinated subpopulation with other people inside this subpopulation), and the minimum routine immunization (RI) coverage with OPV required to eliminate WPV1 transmission from both subpopulations. We adapt a hypothetical population that we previously characterized to illustrate changes in population immunity to transmission during different stages of polio eradication [2] by dividing it into 2 subpopulations (ie, general and undervaccinated). For simplicity in this conceptual discussion, we do not consider supplementary immunization activities (SIAs) or partial RI coverage and instead characterize only RI coverage with exactly 3 OPV doses. If both subpopulations maintain equal coverage, then the model inputs in Table 1 would imply a theoretical threshold RI coverage with 3 OPV doses of approximately 0.92 to eliminate WPV1 transmission for this conceptual population. To characterize the well-vaccinated general population we assume that its RI coverage with 3 OPV doses remains well above this threshold at 0.95. For each combination of size of the undervaccinated subpopulation and its degree of isolation, we search for its minimum RI coverage with 3 OPV doses to eliminate WPV1, using increments of 0.01 (ie, 1% coverage), and we map the 3-way interaction. The model simulates WPV1 elimination in a subpopulation as soon as the effective (ie, infectiousness-weighted) prevalence of WPV1 infections decreases below a threshold of 5 per million people (ie, the transmission threshold), which sets the force of infection for WPV1 at 0 so that no further indigenous WPV1 transmission can occur [2]. This experimentally determined threshold and all other generic model assumptions (ie, those that do not depend on the setting) produced results consistent with the evidence in an extensive model calibration process that considered 9 diverse situations [33]. The model remains fully scalable (ie, it produces different absolute numbers of cases but the same dynamic behavior for different total population sizes) [31], and therefore we focus on the proportion of the undervaccinated subpopulation as compared to the total population, rather than its absolute size.

**Table 1.** Setting-Specific Model Inputs for a Hypothetical Population, Using Generic Model Inputs Published Elsewhere

**Model input**	**Value**	**Notes**
No. of subpopulations	2	1 undervaccinated subpopulation and 1 general population
Initial age distribution		
0–2 mo	0.01	Age groups of ≥5 y from a previously published hypothetical population [2] were split into groups of 5–15 y and ≥15 y, according to the age distribution in “less developed regions excluding China” [75], to accommodate the subsequently developed generically mixing age groups of 0–4, 5–14, and ≥15 y [33]
3–59 mo	0.15	
5–14 y	0.25	
≥15 y	0.59	
Birth rate, births/person/y	0.02	Adopted from previously published hypothetical population [2]
Death rate, deaths/person/y	0.02	Adopted from previously published hypothetical population [2] and applied equally to each age group
Basic reproduction number	10	Adopted from previously published hypothetical population [2]
Proportion of transmission via oropharyngeal route	0.3	Adopted from previously published hypothetical population [2]
Average per-dose OPV take rate for serotype 1	0.5	Similar to typical values in low-income countries for bivalent OPV [33, 34]
Routine immunization coverage with exactly 3 OPV doses in the general population	0.95	Fixed well above the theoretical threshold of 0.92 implied by the other setting-specific inputs from this table

Generic model inputs are from reports by Duintjer Tebbens et al [33] and Thompson et al [34].

Abbreviation: OPV, oral poliovirus vaccine.

## RESULTS

**Figure 1.**
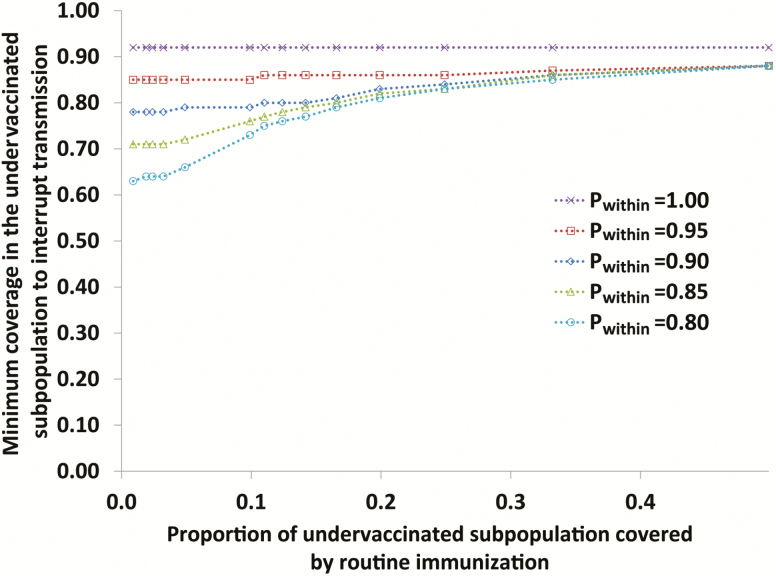
Minimum routine immunization coverage with 3 doses of OPV in the undervaccinated subpopulation required to interrupt serotype 1 wild poliovirus transmission in both subpopulations as a function of (i) the relative size of the undervaccinated subpopulation (y-axis) and (ii) the extent of preferential self-mixing (quantified by *P*_within_, see legend). The model assumes that routine immunization coverage in the general population remains constant at 0.95.

Figure 1 illustrates the interaction between the relative size of an undervaccinated subpopulation; its degree of isolation from the well-vaccinated general population, as indicated by its intensity of self-mixing (p_within_); and the minimum RI coverage in the subpopulation required to eliminate WPV1 transmission. If the undervaccinated subpopulation remains fully isolated from the general population (ie, p_within_ = 1), then it essentially behaves like an island and needs to attain coverage equal to the theoretical threshold of 0.92. If the undervaccinated subpopulation mixes with the general population, then the subpopulation effectively receives some benefits from the high population immunity to transmission in the general population. As a result of the subpopulation’s mixing with the general population, which includes relatively fewer susceptible individuals owing to its assumed RI coverage of 0.95, the mixing effectively reduces the force of infection in the undervaccinated subpopulation, which can help it fall below the transmission threshold. However, even for an undervaccinated subpopulation that is 1/100th the size of the general population (ie, the smallest value represented in Figure 1), for a relatively high degree of mixing with the general population (ie, p_within_ = 0.8, which means that 80% of all contacts occur with people in the same small subpopulation and, thus, that 20% of all contacts by people in the undervaccinated subpopulation occur with people in the general population), the coverage in the undervaccinated subpopulation must still reach at least 63%, or approximately 60% of that in the general population, to eliminate WPV1 transmission. For larger undervaccinated subpopulation sizes, the minimum coverage required to eliminate WPV1 transmission increases and eventually approaches the theoretical threshold of 0.92. Figure 1 suggests that, even with moderate degrees of isolation, pockets of preferentially mixing subpopulations with suboptimal but still relatively high coverage can independently sustain WPV1 transmission despite high population immunity to transmission in the general population. This conceptual behavior highlights the challenge of eradication and explains why undervaccinated subpopulations play such an important role.

Our literature search identified 22 studies, 10 of which we excluded (4 reported epidemiological results; 2 were theoretical studies; 2 were studies of knowledge, attitudes, and preferences; and 2 were not about polio). We reviewed the remaining 12 studies and the references that they included. A prior analysis of polio epidemiological experience showed that >50 countries reported 1 or more annual paralytic polio cases caused by a WPV or cVDPV between 2000 and 2014, which indicated that these countries did not achieve or failed to maintain sufficient population immunity to transmission to stop or prevent transmission [35]. Looking closely at these outbreaks, we see that heterogeneity in vaccination coverage within a country allowed key subpopulations to sustain transmission, and as suggested in Figure 1, some countries with national health systems that perform well on average include undervaccinated subpopulations that can sustain transmission and challenge eradication efforts. In some cases, these weak links only become apparent after cases occur and subsequently trigger a focus of programmatic activities. The insecure areas of Borno (in Nigeria) and the Federally Administered Tribal Areas (in Pakistan) provide clear examples of key subpopulations of undervaccinated individuals. Polio modeling demonstrated the need to explicitly characterize undervaccinated subpopulations in northern India [34, 36], northwest Nigeria [34, 37, 38], Israel [39], the Netherlands [33], and the United States [40] to capture the dynamics of poliovirus transmission in these populations. The strategies required to address undervaccinated subpopulations must consider the specific nature of these subpopulations, including ways to ensure effective identification, communication, and engagement, and good disease surveillance. An analysis of the role of using expanded age groups in SIAs demonstrated the importance of explicit consideration of preferentially mixing undervaccinated individuals [41], as later shown independently by 2 other studies [42, 43]. In the context of the long-term risks for polio, undervaccinated subpopulations also represent a critical focus for long-term risk management. Notably, subpopulations with poor program quality may pose higher risks of cVDPVs and/or inadvertent use of OPV-containing vaccine after homotypic OPV cessation, and inadequate surveillance in these subpopulations would delay response [7, 31].

A close look at some of the outbreaks also revealed that some occurred in countries with disrupted health systems [35]. For example, war-torn areas with unstable governments (eg, Somalia) and poor countries with underserved populations (eg, Haiti) face significant challenges reaching eradication goals, and these governments require support from partners such as the GPEI to achieve goals like polio eradication. Natural disasters (eg, the flooding in Pakistan during 2010) also disrupt systems and pose a risk for outbreaks, and these events similarly require the support of an organization like the GPEI that can mobilize global resources to support action. Polio outbreaks also tragically reveal that even countries with strong health systems can become fragile and need support. For example, the Syrian health system provided immunization with high coverage, and Syria stopped poliovirus transmission prior to 2000, but the recent civil war disrupted the health system, and Syria experienced a WPV1 outbreak in 2013–2014 [44]. These results demonstrate that future efforts to eradicate vaccine-preventable diseases should expect countries with disrupted health systems to represent key subpopulations of concern.

Review of the literature also demonstrated issues associated with competing GPEI objectives that effectively created undervaccinated subpopulations for different serotypes in the case of polio eradication. Although many people think of polio as a single disease, 3 serotypes exist (ie, 1, 2, and 3) that can circulate independently, and eradication requires stopping transmission of all 3 serotypes. The GPEI began to prioritize WPV1 eradication in the mid-2000s, when it encouraged the use of monovalent OPV serotype 1 (mOPV1) in SIAs, based on arguments that competition between the serotypes in tOPV led to vaccine failure for serotype 1 [45]. Unfortunately, the introduction of mOPV1 did not stop WPV1 transmission and created immunity gaps that allowed WPV3 outbreaks. The subsequent use of mOPV3 and later use of bivalent OPV (bOPV; which contains serotypes 1 and 3 OPV) in SIAs led to the apparent disruption of transmission of WPV3 [9]. However, it also effectively created undervaccinated subpopulations for serotype 2 in tOPV-using countries with insufficient RI coverage [36, 37]. The immunity gaps led to a significant increase in cVDPV2s reported after the mid-2000s [46]. These gaps led to the need to increase population immunity to serotype 2 transmission prior to OPV2 cessation by using tOPV, as demonstrated in 2014 to motivate programmatic efforts prior to OPV2 cessation [7] and as shown independently in 2016 [47]. While imperfect vaccine seroconversion implies the need to give multiple doses to successfully immunize children, the failure to vaccinate against all 3 serotypes, not vaccine failure, consistently represented the main obstacle to interrupting transmission. Given that Nigeria historically exported WPV1 to numerous countries in Africa and elsewhere that did not maintain sufficient population immunity to poliovirus transmission [48], countries with poor RI coverage remain at risk. These countries need to continue to conduct preventive SIAs, using bOPV, to keep their population immunity to transmission high and prevent any importations of the circulating WPV1 from restarting transmission and causing new outbreaks within their borders [49]. The increased use of inactivated poliovirus vaccine (IPV), which results in a weaker immune response at the individual level and leads to relatively lower population immunity to transmission than OPV, will not play an important role in preventing cVDPVs in OPV-using countries prior to bOPV cessation [50], substantially accelerate eradication in polio-endemic countries [36–38], or represent a cost-effective option when used in addition to OPV in outbreak response [51]. Children with only IPV-induced protection will effectively represent a different immunological subpopulation, with additional complexity arising if the immunity derives from receipt of 1 or more fractional IPV versus full IPV doses. Future efforts to eradicate vaccine-preventable diseases that use vaccines containing multiple serotypes and/or antigens will need to ensure that focusing on one component does not adversely impact other components.

Finally, the literature suggests that, following OPV cessation, the potential reintroduction of live polioviruses into circulation by iVDPVs poses a threat to a successful polio endgame [27–29, 52–59]. Although effective RI with IPV and outbreak response could mitigate the risks, IPV use alone cannot eliminate the risk, and iVDPVs represent a key subpopulation for ongoing risk management [28]. Efforts to develop antiviral compounds to treat iVDPVs and to screen for iVDPVs represent important strategies to effectively reduce the risks [53]. Recognition of this key subpopulation suggests that future efforts to eradicate vaccine-preventable diseases should consider populations with chronic infections, immunological or other conditions, or other factors that may lead to the need to specifically manage the risks.

## DISCUSSION

In the context of a global eradication effort, high-risk subpopulations represent the weakest links and require significant resources, while all populations must maintain high levels of immunization until global success. Our hypothetical example highlights the importance of attaining high coverage in all populations, because any missed preferentially mixing undervaccinated communities can sustain WPV1 transmission or vaccine-related transmission after OPV cessation. This finding helps to explain the observation of WPV1 and cVDPV2 in the Lake Chad Basin and the border area between Pakistan and Afghanistan. Even after finding missed children in northern India and northwest Nigeria, interrupting transmission required multiple SIAs and specific targeting of unvaccinated and undervaccinated populations, and the same efforts will need occur in northeast Nigeria and the border areas between Pakistan and Afghanistan.

The global capacity created by the GPEI to manage the risks related to key subpopulations that represent weak links for polio eradication currently represents a critical global resource for polio and other infectious diseases. The global polio eradication mandate allows the GPEI to mobilize resources to respond to outbreaks anywhere in the world, including support for response to diseases other than polio. For example, when the severe acute respiratory syndrome outbreak occurred, the global polio laboratory network played a key role in the early response activities and laboratory characterization of the virus. More recently, the GPEI played a critical role in outbreak response to the Ebola virus in Africa and helped contain the Ebola virus importation into Nigeria [60]. With respect to polio, the GPEI demonstrated that it can overcome the challenge of improving access in areas not controlled by the central government and in numerous war-torn areas, including Cameroon, Iraq, Somalia, Sudan, Syria, and Ukraine. In many outbreak situations, the GPEI coordinated the provision of other human services in addition to polio immunization. Providing these services often enabled polio vaccination to occur [61], although offering these services represents a use of GPEI resources that some may perceive as not directly supporting polio eradication. The current global capacity for providing necessary health services in response to health system disruptions represents a resource that could disappear once the GPEI gets disbanded. Our review suggests the need to maintain this type of capacity, which other efforts to eradicate vaccine-preventable diseases will likely need.

With respect to managing the subpopulations created by the use of vaccine that does not contain all of the available serotypes, these issues continue to raise challenges for the GPEI. The GPEI now faces 2 significant threats, from persistent cVDPV2 and WPV1, and can no longer use tOPV as a single tool to combat both [62], despite it representing the best option if available [63]. Thus, protecting against all 3 serotypes in areas with significant fecal-oral transmission requires administration of both mOPV2 and bOPV, including potential coadministration or rapidly alternating SIAs. Clinical trials suggest that interference with OPV2, the least attenuated of the 3 OPV serotypes, leads to lower serotype 1 seroconversion in individuals after 1 dose of tOPV (and by extension mOPV2 coadministered with bOPV) than after 1 dose of bOPV [64–68]. However, these studies also show that this difference essentially disappears after the individuals receive additional doses, with uniformly individual high seroconversion rates measured for all serotypes after 3 tOPV doses in several recent studies in developing countries [65–68].

Transmission depends on unvaccinated and undervaccinated individuals and not on individuals observed in clinical trials. While clinical trials are helpful for understanding individual immunity, they do not provide information about populations, which contain a heterogeneously interacting mixture of individuals who did or did not receive different poliovirus vaccines and live poliovirus exposures, leading to different types and degrees of immunity to poliovirus transmission. Population immunity determines transmission, which depends on the virus finding enough unvaccinated and undervaccinated individuals to infect such that it does not die out. Recent modeling showed almost no difference in population immunity to serotype 1 transmission in northwest Nigeria after repeated bOPV rounds as compared to repeated tOPV rounds [69]. In contrast, giving bOPV and no tOPV resulted in rapidly decreasing population immunity to serotype 2 transmission and unchecked spread of cVDPV2 [69]. For any 1 SIA, only a small fraction of doses given represent first doses, and all outbreak response activities should include a minimum of 3 SIAs [62], so any focus on a single SIA misses the SIA coverage and larger population issues, which ultimately matter because transmission occurs in populations. In the context of few opportunities to gain access to areas with missed children, some may favor the use of bOPV alone instead of coadministration of bOPV and mOPV2 (which is required to deal with the ongoing threat of the persistent cVDPV2), which ignores the population dynamics associated with repeated SIAs. However, without improved access to the undervaccinated population, neither WPV1 nor cVDPV2 will likely die out.

The reality of heterogeneity makes management more complex. In addition, our understanding of subpopulations and heterogeneity remains limited by the quality of the information available and this complicates modeling. In the context of the example presented in this study, we emphasize the simplified approach we use in our deterministic model to approximate the stochastic process of virus die out [33]. In reality, die out of a poliovirus in a small population can occur by chance even with coverage below the theoretical minima presented in this study [70]. However, if this occurs then population immunity to transmission still remains insufficient to prevent imported viruses from reestablishing transmission or to prevent the emergence of indigenous cVDPVs in the event of further reductions in population immunity to transmission.Dealing with heterogeneity can prove essential to obtaining the high levels of program performance required to stop and prevent transmission [71]. While considerable attention continues to focus on strengthening health systems, a key insight emerging from polio eradication relates to the critical need to create expectations for performance [71] and maintain them [72]. Lessons learned from reaching the undervaccinated populations in India revealed the need to identify and reach migrants and nomads, and the strategies developed by the national immunization program in India to reach these individuals included the development of microplans and regular immunization activities, in some areas involving innovative strategies such as using a helicopter to supply vaccines and other health interventions to remote areas. Like Egypt [73], India incorporated surveillance for neonatal tetanus into polio surveillance [74] and translated the lessons from polio eradication into strategies to deliver vaccines and interventions that support its efforts to meet national goals for neonatal tetanus and the elimination of other vaccine-preventable diseases (eg, measles and rubella). Since even strong health systems can experience disruptions, independent of efforts to strengthen health systems, we should expect the need for some sort of global safety net.

## CONCLUSIONS

Eradication efforts for vaccine-preventable diseases need to create performance expectations for countries to identify and reach all people living within their borders with maintained high coverage with appropriate interventions.

## Notes

***Financial support.*** This work was supported by the Centers for Disease Control and Prevention (Cooperative Agreement Number 1U2RGH001913-01).

***Supplement sponsorship.*** This work is part of a supplement coordinated by the Task Force for Global Health with funding provided by The Bill and Melinda Gates Foundation and the Centers for Disease Control and Prevention.

***Potential conflicts of interest.*** Both authors: No reported conflicts of interest. Both authors have submitted the ICMJE Form for Disclosure of Potential Conflicts of Interest. Conflicts that the editors consider relevant to the content of the manuscript have been disclosed.
